# Child/youth, family and public engagement in paediatric services in high‐income countries: A systematic scoping review

**DOI:** 10.1111/hex.13017

**Published:** 2020-01-24

**Authors:** Gagan Gurung, Amy Richardson, Emma Wyeth, Liza Edmonds, Sarah Derrett

**Affiliations:** ^1^ Department of Preventive and Social Medicine University of Otago Dunedin New Zealand; ^2^ Department of General Practice and Rural Health University of Otago Dunedin New Zealand; ^3^ Ngāi Tahu Māori Health Research Unit Department of Preventive and Social Medicine University of Otago Dunedin New Zealand; ^4^ Children’s Health and NICU Southern District Health Board Dunedin New Zealand; ^5^ Department of Women's and Children's Health University of Otago Dunedin New Zealand

**Keywords:** child health, literature review, paediatric services, patient and public engagement, scoping review

## Abstract

**Background:**

Patient and public engagement in paediatric health‐care decision making is under‐researched, and there is a lack of systematically reviewed literature in this area.

**Objective:**

To examine the extent, range and nature of published research investigating the engagement of children/youth, families and the public in paediatric service improvement, to summarize key aspects of the research identified and to identify gaps to help inform future research needs.

**Methods:**

Literature was sought in MEDLINE, EMBASE, PsycINFO and CINAHL. Eligible articles presented research focused on patient, family and public engagement strategies in the paediatric health‐care setting. Two reviewers extracted and charted data and analysed findings using a descriptive numerical summary analysis and a thematic analysis.

**Results:**

From 4331 articles, 21 were eligible. Most were from the United States. The majority of studies were undertaken in hospital settings and used quantitative methods. Various patient and public engagement strategies/interventions were examined, including shared decision‐making tools, questionnaires, youth councils/family advisory groups, patient portals and online networks. Most of the studies examined child/youth/parent satisfaction, with fewer investigating treatment outcomes or service improvement. The majority of studies investigated an engagement strategy at the ‘individual treatment’ level of engagement. Regarding the continuum of engagement, most of the studies were at either the ‘consultation’ or ‘involvement’ stage.

**Conclusion:**

Future research needs to focus on the investigation of engagement strategies delivered in primary care, and the use of more qualitative and mixed methods approaches is recommended. There is a gap in the area of engagement strategies directed towards ‘service design and resources’ and ‘macro/policy’ levels.

## 1 INTRODUCTION

There has been increasing recognition of the need for patient and public engagement in shaping health services.^1^, ^2^, ^3^ The rationale behind enhancing patient and public engagement is to improve the quality of services, improve health service accountability and responsiveness, and improve health outcomes.^1^, ^4^, ^5^

Patient and public engagement is complex. This complexity is evident given the existence of different definitions, theories, strategies of engagement and differences in how engagement strategies are applied in different health‐care settings.^6^, ^7^, ^8^ Barello et al^3^ call for improved consideration of both the ‘roles’ and ‘elements’ of the engagement process. Roles to consider include the ‘engagers’ (eg health professionals and organizations) and those engaging (eg patients and caregivers). The elements of engagement can include the mode, interventions, devices and contexts. Although different guides have been developed with the intention of helping patients and the public engage in health services, there is no consensus about which methods or strategies are the most effective.^4^, ^9^

There are different theoretical models of engagement, most of which specify levels and domains of engagement. Arnstein's^10^ ladder of participation is among the most popular models of participation (the term engagement is used interchangeably with participation in this review). Arnstein^10^ proposed an engagement model with a ladder of ‘Eight Rungs’ with ascending levels of engagement. The first two rungs, ‘Manipulation’ and ‘Therapy’, are considered non‐participation as they relate to persuading citizens to follow existing plans or diverting citizens from important issues.^10^ The next set of rungs are ‘Informing’, ‘Consulting’ and ‘Placation’, and are seen to represent tokenistic forms of participation.^10^ The higher rungs are ‘Partnership’, ‘Delegated Power’ and ‘Citizen Control’, which are intended to represent true engagement.^10^ Although a popular model, criticisms have been made of the linear conceptualization of Arnstein's model.^11^, ^12^, ^13^ There are a number of other models for engagement proposed by different authors,^6^, ^14^, ^15^, ^16^ all informed by Arnstein's earlier work.

Recent models describe engagement as a ‘continuum’ offering a set of choices rather than citizen control as the only ideal goal.^9^ Charles and DeMaio^14^ describe a multidimensional framework based on decision‐making domains, role perspectives and levels of engagement in health‐care decisions. The first dimension refers to types of health‐care decision‐making contexts or domains, ranging from individual‐level treatment to service delivery and organization, and finally to broad system‐level decision‐making contexts.^14^ The second dimension focuses on two alternative role perspectives participants can adopt in health‐care decision making: as users of health services or with a public policy perspective. The third dimension depicts levels of engagement in health‐care decision making, with Arnstein's ladder simplified into three categories: consultation, partnership and lay control.^14^

There is growing recognition of the importance of children's rights, and their need to participate, including health‐care decision making.^17^ Studies suggest that children can provide valuable perspectives on their experience of local health services.^18^ Similarly, families (and/or legal guardians) are involved in their child's care and in turn are able to share their knowledge with professionals, and their participation can provide a more holistic picture of the child and the way services are delivered.^19^ Engagement in paediatric health‐care decision making can be especially complex due to the interaction between three types of participants: children, their parents and health professionals.^20^ Although a number of systematic and narrative reviews have been published in the area regarding various aspects of patient and public engagement more broadly,^17^, ^18^, ^21^, ^22^ there is an absence of consolidated and systematically reviewed literature examining patient and public engagement in paediatric health services specifically.

According to Anderson et al,^23^ ‘scoping studies are concerned with contextualizing knowledge in terms of identifying the current state of understanding; identifying the sorts of things we know and do not know’. Scoping studies are particularly valuable in areas of emerging interest, such as patient and public health‐care engagement.^24^ The goals of this scoping review are to examine the extent, range and nature of research investigating strategies to engage children/youth, families and the public in paediatric service improvement, to summarize key aspects of the research identified and to identify gaps to help inform future research needs.^25^ Specifically, this review aims to: (a) identify peer‐reviewed literature presenting empirical research investigating strategies to promote engagement in paediatric services; (b) describe the range, strengths and weaknesses of engagement strategies empirically examined in the identified publications; and (c) to identify any gaps in knowledge about patient and public participation in paediatric services to help inform future research needs.

## 2 METHODS

We used a framework recommended for conducting scoping studies.^24^, ^25^ This involved the following stages: identifying the research questions; identifying relevant studies; study selection; charting the data; collating, summarizing and reporting results; and consultation.

Additionally, we followed the PRISMA Extension for Scoping Reviews (PRISMA‐ScR) checklist.^26^

### 2.1 Identifying the research questions

To capture a broad range of literature, but at the same time to ensure a manageable volume of literature, we developed research questions which were broad with ‘a clearly articulated scope of inquiry.’^24^ Refined by using the Population, Intervention, Comparison, Outcome, Context (PICOC) question formulation process advocated by the Center for Evidence‐Based Management^27^ (see Supinfo S1), the following review questions were used:

- How have patients, families and the public been engaged in paediatric service improvement activities in high‐income countries?

More specifically, this review aimed to answer:

- What is the extent, range and nature of research related to patient, family and public engagement in paediatric services?
- What are the patient, family and public engagement strategies implemented in paediatric services?
- What are the experiences/outcomes/impact of patient, family and public engagement strategies in paediatric services?

### 2.2 Identifying relevant studies

Four electronic databases (MEDLINE, EMBASE, PsycINFO and CINAHL) were used to capture literature focused on engagement in paediatric services. Different search terms (keywords or phrases) related to the concepts of: (a) patient, family and public engagement, and (b) paediatric health‐care settings were used; then, search strings/terms were developed and used by combining the terms according to ‘Boolean logic’.^28^ The complete list of the terms used to search the four databases (identical or slightly variant versions employed in all databases) is included in Supinfo S2. Where possible, Medical Subject Headings (MeSH) terms were used while searching the databases. However, available MeSH terms were insufficient, so free‐text words were also used.

#### 2.2.1 Inclusion criteria

Guided by the review questions, we included research papers published in peer‐reviewed journals which presented research focused on patient, family and public engagement strategies in the paediatric health‐care setting including the implementation, experience, effectiveness, process or outcomes. Only papers written in the English language and published between January 1990 and 6 December 2017 were included. Additional inclusion criteria included studies conducted in high‐income countries as defined by the World Bank.^29^ We excluded studies not focused on paediatric services. Review articles, discussion papers, think pieces, commentaries and editorials were also excluded. Papers focused on patient, family and public engagement in research projects, paediatric patient satisfaction papers (unless explicitly used by a service to enhance engagement), and papers not available in full text were also excluded.

### 2.3 Study selection

Titles and abstracts of all articles found were uploaded into EndNote to form a database. All the duplicates were then removed. Following this, articles were uploaded into Rayyan QCRI, a web/mobile application that assists with screening, to conduct the remaining review process.^30^

After a review of all titles and abstracts by one reviewer (GG) using the inclusion/exclusion criteria, full‐text versions of all potentially eligible peer‐reviewed papers were accessed. Full‐text articles were independently screened by two reviewers (GG and AR). Any disagreement between them over the eligibility of particular studies was resolved through discussion with a third reviewer (SD).

### 2.4 Data charting

Two reviewers (GG and AR) independently extracted data using a standardized data charting form, and discrepancies were identified and resolved through discussion with third reviewer SD. Extracted information included author(s); title and aims of the study; study methodology (design, methods of data collection and analysis procedure); setting; study population; details of the engagement strategies; and main findings and outcome measures.

### 2.5 Collating, summarizing and reporting findings

A descriptive numerical summary analysis and a qualitative thematic analysis of the papers eligible for inclusion were conducted.^24^ Information for each study regarding aims, setting, design, sample, engagement strategies (types of interventions and/or activities undertaken), primary outcome(s) and main findings was summarized narratively to describe the characteristics of included studies (Table 1). In order to explore the range of engagement strategies implemented to date, studies were further examined and classified using qualitative thematic analysis^24^ according to the levels and continuum of engagement (Table 2; GG and SD). This framework has been adapted from the work of Charles and DeMaio,^14^ Ocloo & Matthews,^16^ Arnstein^10^ and Carman et al.^32^ Levels of engagement utilized in each study were also classified according to whether engagement strategies were targeted at the individual/treatment level, the service design and resource level, or the macro/policy level. For the continuum of engagement, ‘consultation’, ‘involvement’ and ‘partnership and shared leadership’ were used (see Supinfo S3 for operational definitions of these terms). Finally, the settings in which engagement occurred were identified, including whether strategies were implemented in hospitals, primary care settings or the community. This was done to detect gaps in the evidence base regarding types of engagement strategies investigated, levels of engagement targeted and settings in which engagement strategies have been delivered, providing clear opportunities for future research.

**Table 1:** Characteristics of the identified papers reporting research examining patient, family and public engagement in paediatric services

Study ID	Author(s)	Aim	Setting	Design	Sample	Engagement strategies	Primary outcome(s)	Main findings
1.	Abrines‐Jaume et al (2016)^34^	To explore the implementation of shared decision making (SDM) and identify clinician‐determined facilitators to SDM	Four child and adolescent mental health service clinics (inpatient and outpatient), United Kingdom	Qualitative study with completion of plan‐do‐study‐act log books	23 professionals (psychiatrists, psychologists, nurses, family therapists, social workers, play therapists)	Implementation of tools to support SDM	States of implementation and clinician‐determined facilitators to SDM	Professionals experienced three states while attempting to implement SDM: apprehension, feeling clunky and integration into practice. Key clinician behaviours required for SDM included effort implementing SDM, trusting the young person in decision making and flexibility as per child needs
2.	Applegate et al (2003)^37^	To test whether a brief intervention could increase question asking among parents of paediatric diabetes patients and parents of sickle cell patients	A diabetes clinic and a paediatric haematology outpatient clinic at a large teaching hospital, United States	Two experiments: one randomized experiment and one randomized controlled experiment	Experiment 1:62 parent‐child dyads (diabetes); Experiment 2:92 parent‐child dyads (sickle cell) and four paediatricians	Provision of written copies of questions to parents (which served as reminders to ask questions)	Number of questions asked of physician during clinic visit/appointment	Providing parents with written reminders of questions they intended to ask their child's physician increased the number of questions that were asked of the physician. Written reminders were more effective than verbal reminders
3.	Benjamin et al (2015)^36^	The objective of this study was to describe family‐initiated dialogue about medications and health‐care team responses during family‐centred rounds (FCRs) to understand the potential for FCR to foster safe medication use	A tertiary care, academic children's hospital with 61 inpatient beds, United States	Longitudinal descriptive study	150 hospitalized children (mean age of 5.4 y) and their families with at least one video per day of FCR	FCRs, intended to encourage family involvement and participation in the daily presentation of the child's case	Prevalence of family‐initiated medication dialogue during FCR, health‐care team member responses, changes to treatment	Among the 347 videos of FCR reviewed, 132 (38%) contained at least 1 instance of family‐initiated medication dialogue. In response to 8% of instances in which families initiated medication‐related dialogue, a change to the medication treatment plan occurred
4.	Cegala et al (2013)^49^	To test whether parents exposed to a communication skills intervention would participate in a pre‐surgical consultation more than parents in a control group	Paediatric surgical unit of a children's hospital, United States	Randomized controlled trial	65 parents of prospective paediatric surgery patients; intervention arm (n = 63); control (n = 62)	A booklet on the PACE (presenting detailed information about your illness, asking questions, checking your understanding of information and expressing concerns) system. The system includes attention to communication skills: provision of information, asking questions, checking on understanding and expressing concerns.	Participation as indicated by frequencies of parents’ discourse reflecting participation (ie questions and information provision)	Intervention parents participated significantly more than control parents. Intervention parents asked significantly more questions and engaged in significantly more information verifying and expressing concerns. Other significant predictors of parents' participation were consultation length and parents' income
5	Coad et al (2008)^38^	To evaluate the impact of involving young people in developing children's services in an acute hospital trust	One National Health Service (NHS)Trust, United Kingdom	Qualitative evaluation workshop	15 members of a youth council designed to improve children's service delivery (11‐18 y)	A youth council with the aim of influencing trust and health‐care services	Youth council member perspectives on whether their involvement improved children's services, barriers to involvement in service delivery and promotion of young people's involvement in health care	Overall, members felt that they were fully briefed on projects, were helping staff make decisions and that they were contributing to the health care of young people within the trust. All commented that they liked the feeling of being involved and making a difference
6	DeCamp et al (2015)^40^	To evaluate a family advisory board whose parent participants were exclusively limited‐English proficient Latina mothers	Hospital‐based paediatric primary care practice, United States	Qualitative study involving semi‐structured interviews with participants during initial participation and after the final board meeting of the year	10 Latina mothers who participated in the initial year of the family advisory board	Family advisory board where families from a health‐care practice meet with clinical leaders on a regular basis to identify and address areas for improvement	Reasons for joining the board and initial board assessments, assessment of board after the first year of participation, and perceived marginalization and discrimination in health care	Board members expressed a high level of satisfaction with their participation, both during initial participation and after one year. Participants regarded the board as a unique opportunity for Hispanics in the community and felt that board membership countered negative experiences of discrimination and marginalization
7	Emerson et al (2014)^50^	To evaluate the institution of patient satisfaction and safety rounding (‘hourly rounding’) in the paediatric emergency department setting	Urban paediatric emergency department at a tertiary care children's hospital, United States	Prospective observational pre‐post‐study	200 families	Hourly rounding technique delivered in a standardized format	Frequency of call bell activation, patient satisfaction	There was a 50% increase in call bell activation after implementation of hourly rounds (theorized to indicate an increase in communication between the patient and family and the care team). No changes in patient satisfaction were found
8	Heaton et al (2007)^51^	To examine children's, young people's and parents’ access to and use of the English National Health Service Patient Advice and Liaison Service (PALS), and how this could be improved	A mix of acute trusts, children's hospitals and primary care trusts, United Kingdom	Mixed methods: discussion groups and interviews with young people and parents; cross‐sectional postal survey of parents; telephone interviews with PALS staff	30 young people (10‐18 y) and parents – discussion groups; 171 parents – postal survey; 14 PALS staff – telephone interviews	PALS, set up to provide patients and their relatives with a way of obtaining information or expressing concerns about their health care	Awareness of PALS existence and role, access to and use of PALS, effectiveness of and satisfaction with PALS, training of staff	Children and young people were low users of PALS. However, both young people and parents thought that it was a potentially useful service. Parents who responded to the postal survey were generally satisfied with PALS, although approximately half suggested one or more ways in which the service could be improved. Staff thought that PALS advisers would benefit from training in dealing with young people
9	Hussain‐Rizvi et al (2009)^52^	To determine whether parents who deliver asthma treatments in a paediatric emergency department report better adherence to inhaler use at home compared with parents whose children undergo standard care	Paediatric emergency department of an urban public hospital, United States	Randomized control trial	86 children 1 to 5 y of age with an acute asthma exacerbation and their caretakers	Parent administration of asthma treatment	Medication use and delivery methods, child asthma symptoms	Children in the intervention group were 7.5 times more likely to be using the inhaler at a 2‐week follow‐up than children in the standard care group. There were no significant differences in asthma symptoms between the two groups
10	Kelly et al (2017)^35^	To assess parent use and perceptions of an inpatient portal application on a tablet computer that provides information about a child's hospital stay	24‐bed general medical/surgical unit within an 81‐bed tertiary care children's hospital, United States	Mixed methods: portal usage and discharge surveys	296 parents – portal activation; 90 parents – discharge surveys	MyChart Bedside, a patient portal application provided on a tablet computer that allows patients and their families to access real‐time information specific to their hospital stay	Portal usage, portal satisfaction, impact on information needs, engagement, communication, error detection, care safety and quality	The most used and liked features of the portal were vitals, medication list, health‐care team information and schedules. Parent survey respondents were satisfied with the portal (90%), reporting that it was easy to use (98%), improved care (94%) and gave them access to information that helped them monitor, understand, make decisions and care for their child
11	Kelly et al (2017)^53^	To evaluate health‐care team perceptions before and 6 mo after implementation of an inpatient portal application on a tablet computer given to parents of hospitalized children	24‐bed general medical/surgical unit within an 81‐bed tertiary care children's hospital, United States	Repeated cross‐sectional survey study	94 health‐care team members – initial survey; 70 health‐care team members – follow‐up survey	MyChart Bedside	Perceptions of challenges with parents using the portal, impact of portal use on parent and team communication, workload and work satisfaction, care safety and quality	Pre‐implementation, respondents were generally optimistic about the benefits of a portal for parents; however, all anticipated challenges to portal use. Post‐implementation, respondent perceptions of these challenges were significantly reduced
12	Ketterer et al (2013)^54^	To identify the demographic, practice site, and clinical predictors of patient portal enrolment and activation among a paediatric primary care population	Primary care database of an academic children's hospital, United States	Quantitative cross‐sectional analysis of database	84,015 children (mean age of 8.7 y)	MyNemours Patient Portal, a patient portal site that integrates access to personal health information with access to services	Enrolment in MyNemours, activation of MyNemours account, use of MyNemours	Over a 4‐year period, 38% of patients enrolled in the portal; of these, 26% activated the account. There were a number of socio‐demographic disparities in patient portal enrolment
13	Kristensson‐Hallstrom et al (1997)^55^	To assess possible benefits of increasing parental involvement in the care of operated children in a day‐care surgery unit	Department of Paediatric Surgery in a University Hospital, Sweden	Two‐group experimental design (participation in control group followed by participation in intervention group)	88 parents and children (1‐17 y) – control group; 92 parents and children (0.5‐18 y) – intervention group	Individualized information and education about child's post‐operative care	Several indicators of child's recovery from surgery, child's emotional state and co‐operation, parent anxiety, parent ratings of child's pain 3 h post‐surgery	Children of parents in the intervention group started to drink earlier, were mobilized earlier and were discharged earlier than children in the control group. Intervention group children were also rated as being in less pain and fewer children vomited compared with children in the control group
14	Lewis et al (1991)^56^	To design and test a brief educational intervention to promote effective communication between physicians, children and parents during paediatric office visits	Three university‐affiliated general paediatric practices, United States	Randomized controlled trial	141 children (5‐15 y)	Communication intervention designed to enhance communication skills delivered via a brief videotape (individualized for physicians, parents and children)	Medical visit process, information acquisition, child health‐related attitudes and behaviour, satisfaction with communication, child anxiety	Physicians in the intervention group included children in discussions of medical recommendations more often. Children in the intervention group recalled more medication recommendations and reported greater satisfaction. Intervention and control groups did not differ in parent satisfaction, physician satisfaction or child anxiety
15	McGurk et al (2007)^39^	To describe the Treating Patients Well project which sought to enhance staff, user and public involvement in the development of services for neonates and children	Two acute children's wards and a neonatal intensive care unit, United Kingdom	Mixed methods: parent and child satisfaction survey, concept map (graffiti style wall chart)	35 parents and their children (age not available)	The Treating Patients Well project, which aims to promote user involvement and enhance the decision‐making process	Communication, cleanliness, security, process, child's journey through service	Results highlighted a number of potential service improvements, such as addressing the aesthetic appearance of the children's wards and neonatal unit and ensuring that communication with families maintains privacy and dignity
16	Nicholas et al (2007)^41^	To identify perceived outcomes following hospitalized children's participation in a paediatric online support network	Paediatric hospital, Canada	Ethnographic interviews	9 child and adolescent patients (4‐17 y), six parents/family caregivers, three health‐care providers	STARBRIGHT World, an online interactive network for ill children offering information, activities and opportunities for peer networking	Reasons for engaging in the online network, types of network utilization, perceived impacts (benefits and challenges)	The network offered social connection, provided new opportunities and learning, and increased exposure to peers who lived with similar daily realities. Results highlighted a wide 16 spectrum of benefits and challenges in accessing and utilizing the support network
17	Porter et al (2010)^57^	To determine whether a patient‐driven health information technology called ParentLink produced higher‐quality data than documentation completed by nurses and physicians	Site 1: urban children's hospital emergency department; Site 2: a general community emergency department, United States	Quasi‐experimental intervention study where control periods with usual care alternated with intervention periods	1111 parents	Patient‐driven health information technology called ParentLink	Percentage of parent‐child dyads with a valid list of medication allergies and complete emergency department clinical record	Parents’ valid reports of allergies to medications were higher than those of nurses and physicians. ParentLink produced more complete information on illness history than the medical record for five of seven elements
18	Rosati et al (2014)^58^	To examine whether parents of children hospitalized with pneumonia are more satisfied with care when physicians allow them to share decisions on the antibiotic route	Paediatric department of a children's hospital, Italy	Quasi‐experimental intervention study	95 parents of children from 3 mo to 5 y with a diagnosis of severe community‐acquired pneumonia	SDM – parents could choose their child's antibiotic route	Parents' satisfaction with perceived medical information	Of the 18 children's parents in the SDM group, 14 chose the oral antibiotic route mainly to avoid painful injections. Doctors’ explanations were considered better in the SDM group compared with when physicians chose the antibiotic route
19	Taylor et al (2013)^59^	To describe the characteristics of a care coordination programme aimed at supporting families and to compare this programme with provision of a Care Binder	A 430‐bed children's hospital with approximately 50 outpatient care sites, United States	Cross‐sectional survey	75 patients (newborn – 23 y) and parents (50 received the Care Binder only and 25 had access to the Care Coordination Counsellor)	Care Coordination Counsellor programme	Patient/family perception of ‘well‐coordinated care’	Patients/families who received services from the Care Coordination Counsellor reported greater agreement with receiving coordinated care, identifying a key point person for coordination and accessing coordination resources compared with those who received a Care Binder alone
20	Wall‐Haas (2012)^60^	To review the charts of children with asthma who attended a Shared Medical Appointment (SMA) for specific asthma‐related clinical outcomes	Harvard Vanguard Medical Associates Paediatric Department, United States	Pre‐post‐descriptive study	39 children (mean age of 9 y) and parents	SMA, in which four to nine patients and their parents are seen at the same time in a supportive group setting	Asthma‐related clinical outcomes	The SMA had a significant positive impact on asthma‐related outcomes, including use of medication for management, compared with a usual appointment
21	Yager et al (2017)^61^	To evaluate the feasibility and impact of telemedicine for remote parent participation in paediatric intensive care unit rounds	University‐affiliated, 14‐bed paediatric intensive care unit, United States	Proof‐of‐concept study	13 parents	Participation in ward rounds through a mobile teleconferencing unit	Level of satisfaction with remote encounter, communication with staff, reassurance regarding child's care	On average, all participants reported a high level of satisfaction with each telemedicine encounter with minimal disruption. Parents unanimously felt reassured about the care their child was receiving. Some participants were distracted by technical issues that arose

**Table 2:** Number of empirical studies categorized according to the engagement continuum and levels of engagement

Engagement levels	Engagement continuum		
	Consultation Paper ID (n)	Involvement Paper ID (n)	Partnership and shared leadership Paper ID (n)
Individual treatment	2, 7, 12, 14, 17 and 19 (n = 6)	1, 3, 4, 9, 10, 11, 13, 18, 20 and 21 (n = 10)	16 (n = 1)
Service design and resources	8 and 15 (n = 2)	5 and 6 (n = 2)	
Macro policy/strategic			

### 2.6 Consultation

Preliminary findings of the review were shared with key stakeholders, including academics and end users (such as policy and health managers) at a University of Otago symposium to increase the relevance of the findings.^33^ The findings were well‐received by the audience, and their recommendation to include additional references in the discussion of this manuscript was adopted.

## 3 RESULTS

### 3.1 Literature search

**Figure 1:**
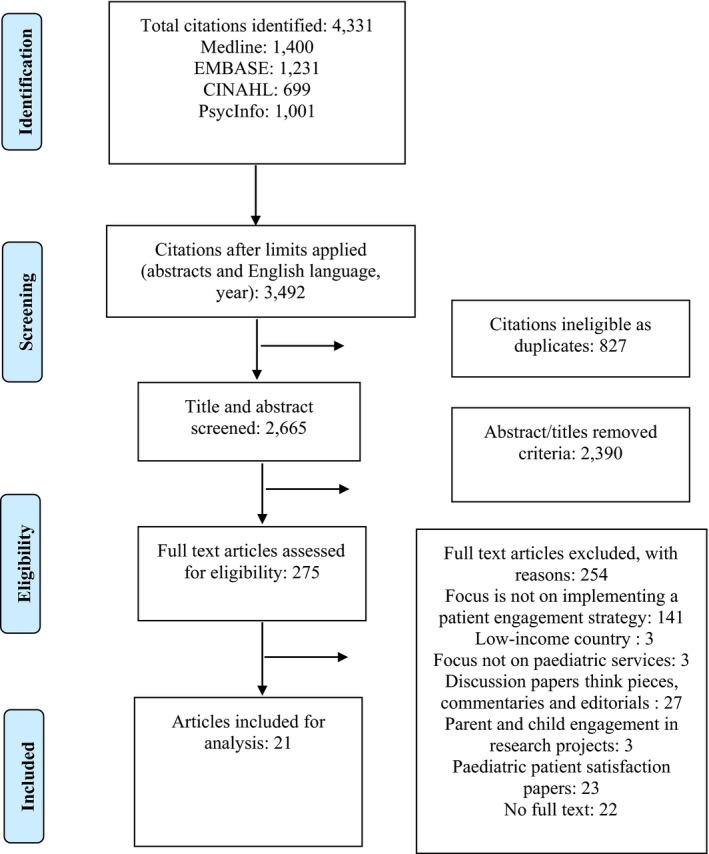
PRISMA Flow Diagram of literature search^48^

A total of 4331 citations were initially obtained. After restricting the search to the availability of an abstract and to English language sources, 3492 citations remained, of which 827 were removed as they were duplicates. After title and abstract review of the remaining 2665 papers, 275 papers remained. Of these, 21 met the inclusion criteria after full‐text reading and data extraction (Figure 1). We summarized an overview of the principal findings for each paper (Table 1). When relevant, we cited either the paper ID from Table 1 or cited the paper directly.

### 3.2 Study characteristics

Of the 21 papers reviewed, 19 were published in or after 2000. In terms of geographical distribution, most papers came from the United States (n = 14), followed by the United Kingdom (n = 4), Sweden (n = 1), Canada (n = 1) and Italy (n = 1; Table 1). No papers reported research undertaken in Australasia (ie Australia or New Zealand). Most papers (n = 20) reported findings of research undertaken solely in hospital settings. Of 21 papers identified, 14 reported methods which were quantitative (2, 3, 4, 7, 9, 11, 12, 13, 14, 17, 18, 19, 20 and 21), four were qualitative (1, 5, 6 and 16), and three were mixed methods (8, 10 and 15). The most common research design employed within the quantitative studies was experimental (2, 4, 9, 13, 14, 17 and 18).

Across the studies, a range of participants were included: parents (n = 7; 4, 6, 7, 10, 17, 18 and 21), parents and their children (n = 6; 3, 9, 13, 15, 19 and 20), children/youth (n = 3) (5, 12 and 14), parents and professionals (n = 1; 2), professionals (n = 2; 1 and 11) and parents, children/youth and professionals (n = 2; 8 and 16).

Various patient and public engagement strategies/interventions, both traditional engagement techniques and new strategies, were used, including shared decision‐making tools (n = 2; 1 and 18), questionnaires (n = 1; 2), youth councils/family advisory groups (n = 2; 5 and 6), patient portals (n = 4; 10, 11, 12 and 17), online networks (n = 1; 16), communication interventions via videotape or booklet, and individualized information and education session (n = 3; 4, 14 and 13), among others (n = 8; 3, 7, 8, 9, 15, 19, 20 and 21).

Regarding outcome measures, most of the studies examined child/youth/parent satisfaction with care after implementation of an engagement strategy (n = 8; 6, 7, 8, 10, 11, 14, 18 and 21) or experiences of participation (n = 5; 1, 4, 12, 16 and 19), including participants’ perceptions of involvement in decision making, communication, implementation experience, etc. Fewer examined whether the implementation of the strategy produced changes in treatment outcomes (n = 4; 3, 9, 13 and 20) and service improvement (n = 4; 2, 5, 15 and 17).

### 3.3 Continuum of engagement

The majority of studies included in this review (n = 17) investigated an engagement strategy at the ‘individual treatment’ level of engagement (Table 2). Few studies (n = 4; 5, 6, 8 and 15) were found at the ‘service design and resources’ level. No study identified in the review investigated a strategy at the macro ‘policymaking’ level. For example, one study exploring the implementation of shared decision making (SDM) and clinician‐determined facilitators of SDM was categorized at the individual treatment level as it focused on SDM at the level of the individual patient‐health professional interaction.^34^ Other examples of ‘individual treatment’ level engagement studies include those focused on questions asked in the individual patient's outpatient appointment, care of the individual child, patient portals and online networks.^35^, ^36^, ^37^ An example of a study focused on engagement at the level of service design and resources investigated the impact of involving young people through a youth council in developing children's services in a hospital trust where youth were involved in activities, ranging from developing patient bedside booklets to advising on menus and websites.^38^ Other examples include involvement in activities such as brochure development, and obtaining patients’ and parents’ perspectives on cleanliness and privacy of services.^39^, ^40^

Regarding the continuum of engagement, most of the studies (n = 20) were categorized to the ‘consultation’ or ‘involvement’ stages. Only one study^41^ was identified as existing at the ‘partnership and shared leadership’ stage of the continuum (Table 2). Consultation represents the weakest form of engagement as it provides an opportunity for patients/public to express their views/opinions, or receive information, but with no guarantee their views will be acted upon. Applegate et al^37^ conducted a study to test whether a brief intervention could increase question asking among parents of paediatric diabetes patients and parents of sickle cell patients. When we analysed the continuum of engagement, we identified this approach as ‘consultation’—as parents were asked what questions they had for the clinicians and were encouraged to ask these either orally or by having their questions written on cards. They were not asked what mattered most to them as patients/parents. They were not asked about their experience of asking the questions. Altogether, eight studies were categorized at the consultation stage of the engagement continuum (2, 8, 7, 12, 14, 15, 17 and 19).

The ‘involvement’ category included papers where the patients and the public expressed their views or were involved in the decision‐making process by giving advice, but without final decision‐making authority. Twelve studies were categorized as being at the involvement stage on the continuum (1, 3, 4, 5, 6, 9, 10, 11, 13, 18, 20 and 21). For example, Benjamin et al^36^ studied family‐initiated dialogue about medications and health‐care team responses during family‐centred rounds (FCRs) to understand the potential for FCR to foster safe medication. We categorized the engagement process in this study as involvement as this was looking for child/parent questions and/or discussion in videoed FCRs leading to a change in treatment for the patient.

‘Partnership and shared leadership’ is characterized by shared power and responsibility, with patients and public as active partners in defining agendas and making decisions. One study was categorized in this stage where engagement occurred through an online network that children/youth could engage with if they wished to.^41^

## 4 DISCUSSION

This scoping review identified 21 studies implementing or evaluating a specific strategy to promote engagement of children/youth, family or the public in paediatric health service improvement activities. To the best of our knowledge, this is the first systematic scoping review of patient and public engagement in the paediatric setting. Our review found an absence of studies reviewing strategies targeting engagement with children/youth and families at the ‘service design and strategy’ and ‘policy‐making’ levels.

Past reviews have reported that literature on public participation in policymaking and priority setting and resource allocation is growing.^42^, ^43^ Gregory,^9^ however, reported in a literature review of consumer engagement in health policy in Australia that engagement has focused on individual participation in health‐care decision making despite the public's desire to participate in planning health services and health‐care priority setting. The majority of studies included in our review also investigated an engagement strategy at the ‘individual treatment’ level of engagement. Although there are well‐regarded patient and public engagement models/frameworks depicting the levels of engagement available, few papers identified in our review reported their findings in relation to such models/frameworks. As the literature suggests, children can provide valuable perspectives on their experience of local health services^18^ and families involved in their child's care can share important knowledge with professionals, providing a more complete picture of the child and the way services should be delivered.^19^ Therefore, directing engagement efforts to design and policy levels is an important avenue for future research.

Regarding the continuum of engagement, most of the studies were found at the ‘consultation’ or ‘involvement’ stages of the continuum and only one at the ‘partnership and shared leadership’ stage. This finding is consistent with past studies of engagement more generally. A scoping review of community participation in rural health identified very few examples of high‐level participation with evidence of partnership, delegated power and citizen control.^44^ Many other studies showed progress to achieve greater engagement is slow and patchy, and often concentrated at the lower levels of the continuum.^16^, ^45^, ^46^ Furthermore, others have found professionals and organizations can, in practice, control the decision making in many contexts.^6^ However, the goal is not always to move to a higher level of the continuum. There is wide variation in preferences for participation in decision making which can be determined by capabilities, resources, types of services/cares and the needs of the particular decision‐making situation.^6^, ^16^, ^32^, ^47^ Hence, it is important to consider the preferences and willingness of patients and the public to participate, with efforts directed away from tokenistic strategies towards partnership approaches to participation. Our review suggests studies also tended to explore strategies that were implemented in the hospital context. There appears to be an absence of studies that aim to promote children/youth and family engagement in other health‐care contexts, for example primary care.

The majority of included studies employed a quantitative design; only a few studies used mixed methods to evaluate engagement strategies. While past reviews have found research in patient and public engagement to be dominated by qualitative methods,^4^ fewer qualitative studies in our review might be due to our inclusion criteria. One of our inclusion criteria was that the papers reported research focused on the implementation, effectiveness, process and outcomes of patient engagement strategies, which resulted in a significant number of intervention studies. More qualitative research would be beneficial to explore mechanisms behind any significant effects found. Such research would also allow participants to describe their experiences, provide suggestions for improvements and identify potential facilitators and barriers associated with different types of engagement strategies.

Most studies we identified examined child/youth/parent satisfaction with care after the implementation of an engagement strategy. Fewer examined whether the implementation of the strategy produced changes in treatment outcomes and service improvement. Systematic reviews conducted by Mockford et al^2^ and Crawford et al^4^ also noted a lack of evidence regarding the impact of participation in general. The purpose/rationale of the engagement strategies can also influence what outcomes/impacts are sought. Engagement efforts can be directed towards achieving intrinsic goals (engagement for the democratic process, accountability, etc) rather than focusing on specific health outcomes.^42^ The gap in evidence of engagement in health service development and improved health outcomes needs to be addressed, especially for communities who may not be as well‐engaged in existing health services.

The results of our scoping review are subject to certain limitations. First, the emphasis of the scoping review on peer‐reviewed publications only may have missed patient and public engagement studies reported in grey literature. It would be worthwhile to further investigate the grey literature, given its potential to identify additional engagement strategies employed in the paediatric context. Secondly, there was limited discussion about the continuum of engagement found in the identified papers. Hence, our categorization of the studies across the continuum needs to be treated with caution. However, the research team made genuine efforts to analyse the studies as accurately as possible. We used clearly defined guidelines to categorize the studies along the continuum, and the analysis was conducted by two researchers independently. Any disagreement between them regarding the eligibility of particular studies was resolved through discussion with the research team. In the context of the apparent literature gap between the availability of theoretical literature regarding depth or continuum of engagement and the application of such a framework in empirical studies, our attempt to categorize studies across the continuum is a strength of the review.

## 5 CONCLUSION

This review found that literature related to patient and public engagement in paediatric services has mostly emerged since the year 2000, and with a hospital and quantitative focus. Studies tended to investigate an engagement strategy at the ‘individual treatment’ level, and most studies were at ‘consultation’ or ‘involvement’ stages of the continuum. We suggest future studies investigating engagement strategies be undertaken in primary care and that qualitative and mixed methods approaches may help strengthen our understanding of engagement mechanisms. Additional studies are needed to investigate the implementation and effectiveness of patient, family and public engagement strategies in the following areas: co‐design of paediatric services; development of policies related to paediatric services; feedback mechanisms to paediatric services; and approaches to improving the experiences of children and their families. Furthermore, the use of theoretical frameworks to measure the engagement process is recommended, with a focus on service design, resources and macro/policy levels.

## CONFLICT OF INTEREST

None of the authors declared any conflicts of interest.

## Data Availability

Data sharing is not applicable to this article as no new data were created or analysed in this study.
