# Supplementary material for: Child/youth, family and public engagement in paediatric services in high‐income countries: A systematic scoping review
Source: Health Expect. 2020 Jan 24;23(2):261–73. doi: 10.1111/hex.13017 (PMC7104655; doi:10.1111/hex.13017)
Supplement: Supplementary file 1 [file HEX-23-261-s001.docx]

***Supplementary File 1* *PICOC***

Population: Patients, families and the population engaged in paediatric service improvement activities

Intervention (phenomenon of interest): Patients, families and the population engagement strategies

Comparison/Control: Not applicable

Outcome: implementation/experience/effectiveness/process/outcomes of patient, families and the population engagement strategies

Context: Paediatric service (e.g. in hospital, clinic, primary care, community or home settings); high-income countries as defined by the World Bank.
