# Supplementary material for: Child/youth, family and public engagement in paediatric services in high‐income countries: A systematic scoping review
Source: Health Expect. 2020 Jan 24;23(2):261–73. doi: 10.1111/hex.13017 (PMC7104655; doi:10.1111/hex.13017)
Supplement: Supplementary file 2 [file HEX-23-261-s002.docx]

***Supplementary File 2. Search terms used in literature review***

| Data bases |  | Key words |
| --- | --- | --- |
| Patient and public engagement | EMBASE | social participation [MeSH] OR community participation OR public participation OR citizen* participation OR lay participation OR user* participation OR consumer participation OR patient participation [MeSH] OR community consultation OR public consultation OR community involvement OR public involvement Or consumer engagement OR community engagement OR patient engagement OR citizen* engagement OR family engagement OR family involvement OR family consultation OR patient experience OR patient and public involvement OR patient and public engagement OR patient and public participation or child* engagement OR child* involvement OR child* experience OR young people participation OR young people engagement |
|  | Medline | social participation[MeSH] OR community participation [MeSH] OR public participation OR citizen* participation OR lay participation OR user* participation OR consumer participation OR patient participation OR community consultation OR public consultation OR community involvement OR public involvement Or consumer engagement OR community engagement OR citizen* engagement OR public engagement OR family engagement OR family involvement OR family consultation OR patient experience OR patient and public involvement OR patient and public engagement OR patient and public participation Or patient engagement OR patient participation[MeSH] OR child* participation OR child* engagement OR child* involvement OR child* experience OR young people participation OR young people engagement |
|  | PsycInfo | social participation OR community participation OR public participation OR citizen* participation OR lay participation OR user* participation OR consumer participation OR patient participation OR community consultation OR public consultation OR community involvement[MeSH] OR public involvement Or consumer engagement OR community engagement OR citizen* engagement OR patient engagement OR patient involvement OR family engagement OR family involvement OR family consultation OR patient experience OR public engagement OR patient and public engagement OR patient and public involvement or patient participation or child* participation OR child* engagement OR child* involvement OR child* experience OR young people participation OR young people engagement or parental involvement[MeSH] OR parent participation OR parent involvement OR parent engagement |
|  | CINAHL | social participation [MeSH] OR community participation OR public participation OR citizen* participation OR lay participation OR user* participation OR consumer participation[MeSH] OR patient participation OR community consultation OR public consultation OR community involvement OR public involvement OR consumer engagement OR patient and public engagement OR patient engagement OR patient and public involvement or public engagement OR patient and public involvement or patient participation or child* participation OR child* engagement OR child* involvement OR child* experience OR young people participation OR young people engagement or parent involvement or parent participation or parent involvement or parent engagement or family engagement OR family involvement OR family consultation OR patient experience |
| Paediatric services | Ovid EMBASE | child health care[MeSH] OR paediatrics[MeSH] OR pediatric hospital[MeSH]OR pediatric ward [MeSH] OR Pediatric nursing [MeSH] OR paediatric outpatient* OR pediatric outpatient* OR child care[MeSH] |
|  | Medline | pediatrics [MeSH] OR child health services[MeSH] OR pediatric care OR paediatric care OR child health[MeSH] OR paediatric* OR pediatric ward* OR paediatric ward* OR child* ward* OR paediatric department* OR paediatric unit* OR pediatric department* OR pediatric unit* OR pediatric hospital[MeSH] OR paediatric hospital* OR child* clinic OR child* clinics OR child* health cent* OR child* health clinic* OR child* welfare clinic* OR child* hospital OR child* hospitals OR children institution OR paediatric health cent* OR pediatric health cent*OR pediatric cent*OR paediatric cent* OR pediatric clinic* OR paediatric clinic*OR community paediatric*OR community pediatric* OR paediatric practice* OR pediatric practice* OR paediatric research OR pediatric research OR paediatric service* OR pediatric service* OR pediatry OR paediatry OR social pediatry OR paediatric health care OR pediatric health care OR pediatric nursing[MeSH] OR paediatric nursing* OR paediatric outpatient* OR pediatric outpatient* |
|  | PsycInfo | pediatrics[MeSH] OR child health services OR pediatric care OR paediatric care OR child* health OR paediatric* OR pediatric ward* OR paediatric ward* OR child* ward* OR paediatric department* OR paediatric unit* OR pediatric department* OR pediatric unit* OR pediatric hospital* OR paediatric hospital* Or child*clinic* OR child* health cent* OR child* health clinic* OR child* welfare clinic* OR child* hospital* OR paediatric health cent* OR pediatric health cent*OR pediatric cent*OR paediatric cent* OR paediatric clinic* OR pediatric clinic* OR paediatric clinic*OR community paediatric*OR community pediatric* OR paediatric practice* OR pediatric practice* OR paediatric research OR pediatric research OR paediatric service* OR pediatric service* OR pediatry OR paediatry OR paediatric health care OR Pediatric health care OR pediatric nursing* or paediatric nursing* OR paediatric outpatient* OR pediatric outpatient* OR child care[MeSH] |
|  | CINAHL | pediatrics[MeSH] OR child health services[MeSH] OR pediatric care[MeSH] OR pediatric ward* OR paediatric ward* OR paediatric department* OR paediatric unit*[MeSH] OR pediatric department* OR child* clinic OR child* clinics OR child* health cent* OR child* health clinic* OR child* welfare clinic* OR child* hospital OR child* hospitals OR paediatric health cent* OR pediatric health cent*OR pediatric cent*OR paediatric cent* OR pediatric clinic* OR paediatric clinic*OR community paediatric*OR community pediatric* OR paediatric practice* OR pediatric practice* OR paediatric research OR pediatric research OR paediatric service* OR pediatric service* OR paediatric health care OR pediatric health care OR pediatric nursing[MeSH] OR paediatric outpatient* OR pediatric outpatient* |
