# Supplementary material for: Child/youth, family and public engagement in paediatric services in high‐income countries: A systematic scoping review
Source: Health Expect. 2020 Jan 24;23(2):261–73. doi: 10.1111/hex.13017 (PMC7104655; doi:10.1111/hex.13017)
Supplement: Supplementary file 3 [file HEX-23-261-s003.docx]

***Supplementary file 3. Definition of levels and continuum of engagement^1-4^***

| **Levels of engagement** | **Continuum of engagement** |
| --- | --- |
| Three levels of engagement were used for the study: treatment, service design and resources, and macro policy/strategic level. Treatment level refers to integration of patients and public viewpoints/perspectives on decision making related to prevention, diagnosis, and treatment, including managing patient’s health to be provided to patients/individuals. The second service design and resources relates to inclusion of patients and public viewpoints/perspectives on decision making related to resource allocations and service design of health care organisations such as hospitals and clinics. At the macro policy/strategic level, engagement focuses on involvement of patients and public perspectives on developing/evaluating policy and programme at national, state or even local level. | Patient and public engagement activities can range along a continuum, from consultation to partnership and shared leadership. At the continuum lower end, patients and public are involved but have limited power or decision-making authority. Specifically speaking, consultation represents the lowest form of participation in decision making which provides an opportunity for patients/public to express their views/opinions, but there is no guarantee that their views will be taken into action. Examples include patients receive information, participate in a survey or focus group. Involvement relates to the middle point of the continuum with patients and public express their views and are involved in decision making process by giving advice but they have no final says in decision making. Examples include patients/public are involved in an advisory board or they provide recommendations. Partnership and shared leadership are characterised by shared power and responsibility, with patients and public as active partners in defining agendas and making decisions. Examples include treatment decision making made jointly by patient and clinician, co-design of health services etc. |

1. Arnstein SR. A ladder of citizen participation. *Journal of the American Institute of Planners.* 1969;35(4):216-224.

2. Carman KL, Dardess P, Maurer M, et al. Patient and family engagement: a framework for understanding the elements and developing interventions and policies. *Health Affairs.* 2013;32(2):223-231.

3. Ocloo J, Matthews R. From tokenism to empowerment: progressing patient and public involvement in healthcare improvement. *BMJ Quality & Safety.* 2016;25(8):626-632.

4. Charles C, DeMaio S. Lay participation in health care decision making: a conceptual framework. *Journal of Health Politics, Policy and Law.* 1993;18(4):881-904.
